# Association of Radiation Doses and Cancer Risks from CT Pulmonary Angiography Examinations in Relation to Body Diameter

**DOI:** 10.3390/diagnostics10090681

**Published:** 2020-09-09

**Authors:** Hanif Haspi Harun, Muhammad Khalis Abdul Karim, Zulkifly Abbas, Mohd Amir Abdul Rahman, Akmal Sabarudin, Kwan Hoong Ng

**Affiliations:** 1 Department of Physics, Faculty of Science, Universiti Putra Malaysia, Serdang 43400, Selangor, Malaysia; hanifhaspi@gmail.com (H.H.H.); za@upm.edu.my (Z.A.); mohdamir@upm.edu.my (M.A.A.R.); 2 Department of Radiology, Hospital Kuala Lumpur, Jalan Pahang, Kuala Lumpur 50586, Malaysia; 3 Department of Diagnostic & Applied Health Sciences, Faculty of Health Sciences, Universiti Kebangsaan Malaysia, Kuala Lumpur 56000, Malaysia; akmal.sabarudin@ukm.edu.my; 4 Department of Biomedical Imaging, Faculty of Medicine, Universiti Malaya, Kuala Lumpur 50603, Malaysia; ngkh@ummc.edu.my

**Keywords:** radiation dose, cancer risk, CTPA examinations, body diameter, SSDE

## Abstract

In this study, we aimed to estimate the probability of cancer risk induced by CT pulmonary angiography (CTPA) examinations concerning effective body diameter. One hundred patients who underwent CTPA examinations were recruited as subjects from a single institution in Kuala Lumpur. Subjects were categorized based on their effective diameter size, where 19–25, 25–28, and >28 cm categorized as Groups 1, 2, and 3, respectively. The mean value of the body diameter of the subjects was 26.82 ± 3.12 cm, with no significant differences found between male and female subjects. The risk of cancer in breast, lung, and liver organs was 0.009%, 0.007%, and 0.005% respectively. The volume-weighted CT dose index (CTDI_vol_) was underestimated, whereas the size-specific dose estimates (SSDEs) provided a more accurate description of the radiation dose and the risk of cancer. CTPA examinations are considered safe but it is essential to implement a protocol optimized following the As Low as Reasonably Achievable (ALARA) principle.

## 1. Introduction

Computed tomography (CT) scanning has become the most popular imaging technique and the number of exams using this technique is steadily increasing. Despite its good diagnostic value for disease visualization, the International Commission for Radiological Protection (ICRP) expressed its concern about the use of CT in advanced medicine and the fact that it can cause cancer risk relative to other imaging methods [1]. CT Pulmonary Angiography (CTPA) is one of the imaging techniques that enables the visualization of pulmonary arteries and the diagnosis and treatment of pulmonary embolism (PE). PE is considered a significant health condition associated with high mortality and it requires rapid and accurate diagnosis, particularly in patients at high risk. More than 90% of appropriate PE tests can be achieved through the development of CT technology [2]. However, a single CTPA examination can contribute up to 10 millisievert (mSv) of effective dose (*E*), which increases the risk of radiation-induced cancer to populations [2,3].

Volume-weighted CT dose index (CTDI_vol_) typically represents the standard dosimetry for CT scanners based on the standardized poly-methyl methacrylate (PMMA) phantom dose calculation with pitch value consideration [4]. CTDI_vol_, however, has many potential flaws as it does not consider the size of the body, which varies between patients, especially children. In 2011, the American Association of Physicists in Medicine (AAPM) introduced Size-Specific Dose Estimates (SSDE), which incorporate individual patient characteristics into the estimation of CTDI_vol_ [5]. Instead of basing dose calculation solely on a phantom, SSDE requires the input of individual patient size in the CT scanner [6]. SSDE′s relationship with CTDI_vol_ was found to be inversely proportional to the patient′s size. When the size of the patient increases, the ratio decreases, which improves sensitivity in dose calculation [7]. Radiation exposure is a crucial issue due to an increase in the risk of inducing cancer, especially in younger patients [8,9]. More than 2% of the population receives a significant number of doses and those populations are at risk of developing cancer, where younger patients are the main contributor to that statistic [2,10]. Therefore, strategies to reduce CT dose while maintaining good image quality are a major focus of researchers in the field.

Estimation of organ dose and cancer risk according to the body habitus is precise and accurate. Both assessments vary in different conditions, depending on the age, sex, and population studied [11]. The limitation of radiation dose estimation may be overcome by assessments tailored to individual patient exposure rather than to a general population. The seventh report on Biological Effects of Ionizing Radiation (BEIR VII Phase 2) by the United States National Academy of Sciences introduced cancer-risk estimates in radiological scans. Previous research indicated that the harmful effects of exposure to radiation in vulnerable patient populations, in particular young women and children, are higher [12]. It is important to determine an organ-equivalent dose before achieving an estimation of the risk of cancer. Awareness of the possible radiation risk will allow radiology staff to be more prepared and more likely to encourage improvement in each CT scanning examination. Therefore, this study aimed to evaluate the dose exposure and to estimate the attributes of cancer risk from CTPA examination based on patients’ size and habitus.

## 2. Materials and Methods

### 2.1. CT Parameter Measurements

This study was approved by the Medical Research and Ethics Committee (MREC) of the Ministry of Health Malaysia (MOH), which waived patient consent forms for the retrospective analysis with the approval ID; NMRR-18-3088-44138, dated 13 March 2019. From September 2018 to February 2019, reports of 100 adults undergoing CTPA exams were obtained from Kuala Lumpur Hospital, Malaysia. The scanning was performed using Philips Brilliance (Phillips, NL, USA) 128-slice CT scanner, and the images were reconstructed automatically.

During examinations, 40 to 70 mL of iodinated contrast medium, followed by 50 mL saline, were intravenously injected into the subjects at a flow rate of 5 mL/s. The bolus tracker technique was performed by placing the region of interest (ROI) on the main pulmonary trunk. The scan was started after 3 to 14 s with a threshold set at 70 Hounsfield units (HU). The scanning was performed craniocaudally using active Z-dose modulation with 100/120 kVp tube potential, 0.798 pitch factor, and 0.625 × 40 mm beam collimation size. Images were reconstructed with a slice thickness of 1 mm and a matrix size of 512 × 512. The iDose^4^ level 4 iterative reconstructive technique for post-processing of images was selected to improve the CT images.

**Figure 1:**
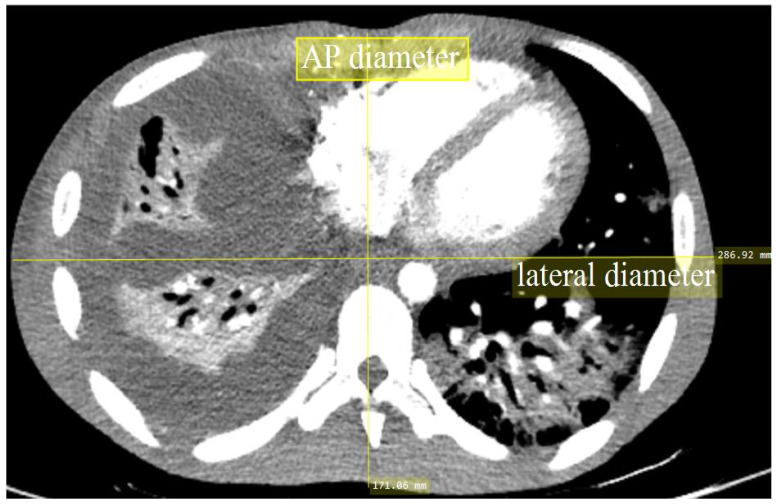
Patient′s size measurement at the mid-slice location of the transverse CT images.

All acquisition parameters data, such as tube voltage (kVp), tube current (mA), rotation time, pitch factor, CTDI_vol_, and dose-length product (DLP), were obtained from the CT console and recorded into a standardized form. The anteroposterior (AP) and lateral (LAT) lengths of each subject’s image were measured using digital calipers on the scanner console at the mid-slice location of the transverse CT images, as illustrated in Figure 1. Only the scan data of pulmonary embolism scans were included, whereas cases with incomplete details and modified protocols were excluded.

### 2.2. Radiation Dose

CTDI_vol_, AP, and LAT lengths from the scanner console were used to measure SSDE value. SSDEs were also measured using CT-EXPO Ver 2.3.1 (Germany) based on the scanning parameter used in each examination for comparison purposes. SSDEs were estimated by adopting the AAPM report. A total of 220 reports and the cross-sectional area of the subjects′ body were estimated by using the following equation [13]:

(1) Cross-sectional area, σ=LAT+AP

where LAT and AP represent the length in cm for lateral and anteroposterior, respectively. The cross-sectional area was used to consider the conversion factor $f_{D_{W}}$ based on the water equivalent diameter, *D_W_*_._ *D_W_* is a size metric that considers both geometric size and patient′s X-ray attenuation factor, and shown in the following equation:

(2) $$D_{W}=2\sqrt{\left[\frac{1}{1000}\bar{HU}+1\right]}\frac{A}{\pi }$$

where $\bar{HU}$ is a mean HU in the ROI of cross-sectional images and *A* is the area in pixel value, px^2^. Next, the SSDE was obtained by multiplying the normalized *D_W_*, $f_{D_{W}}$ with the estimated CTDI_vol_:

(3) $$SSDE=f_{D_{W}}\times CTDI_{vol}$$

In this study, *E* and organ dose such as breast, lung, and liver were estimated by using CT-EXPO software. This software offers automatic output calculation of radiation exposure to the organs based on a detailed scanner model with manufacturer and scanning parameters. The software used a Monte Carlo simulation model and estimated the radiation dose based on radiation transport attributable organ dose on the adult phantom.

### 2.3. Risk Assessments

One of the main contributions of dose output is the estimation of the probability of radiation-induced risk. Hence, the cancer risk for selected organs was estimated using the following equation:

(4) $$\mathrm{Cancer}\;\mathrm{risk},\;R=\sum r_{T}.H_{\begin{array}{l} T \end{array}}$$

where $r_{T}$ is the nominal risk factor attained from the International Commission on Radiological Protection (ICRP) Publication 103 (ICRP 2007) and $H_{T}$ is the organ-specific equivalent dose estimated using CT-EXPO.

### 2.4. Statistical Analysis

Data were analyzed using IBM SPSS version 25.0 (IBM Corporation, Armonk, NY, USA). Data are presented in descriptive analysis and expressed as mean ± standard deviations. The Shapiro–Wilk test was used to determine the normality of the data. Differences between the two groups were determined using the Mann–Whitney test and the Kruskal–Wallis test was used for more than two groups (*p* < 0.05). A *p*-value of <0.05 was chosen to indicate statistically significant differences. 

## 3. Results

**Figure 2:**
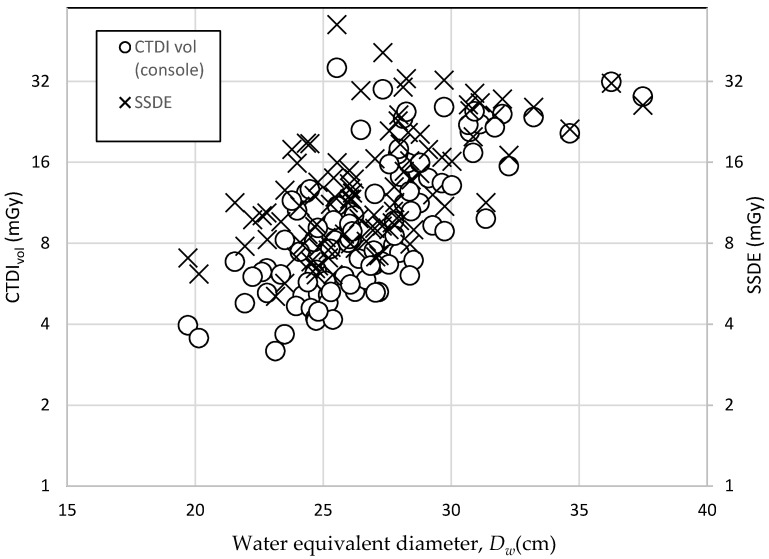
Relationship between CTDI_vol_ and SSDE with *D_W_* of the subjects.

**Figure 3:**
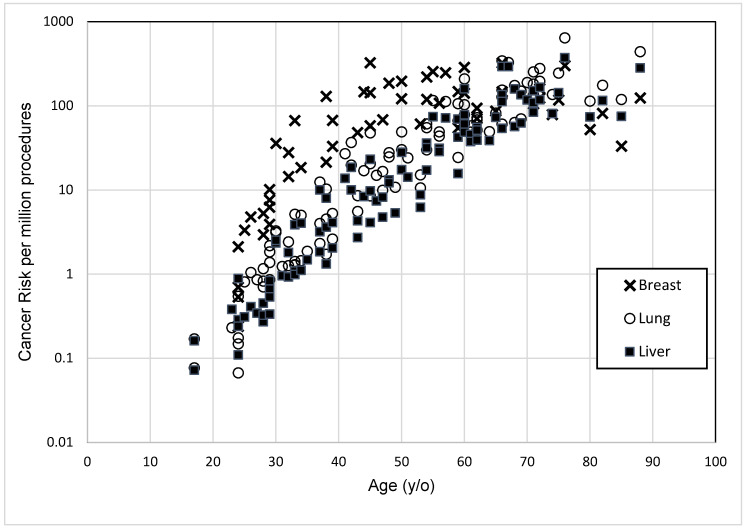
Relationship between cancer risk per million procedure with the age of the subjects.

Table 1 shows the mean baseline characteristics of study subjects comprised of 42 men and 58 women. The *D_W_* ranged from 20.14 to 37.48 cm in men and 19.71 to 32.25 cm in women. The calculations of CTDI_vol_, DLP, *E*, and organ dose were grouped according to *D_W_* and the total was calculated, as indicated in Table 2. The mean values obtained for CTDI_vol_, DLP, and *E* values were 11.06 ± 7.1, 400.38 ± 259.10, and 8.68 ± 5.47 respectively. The mean values of organ dose for breast (women only), lung, and liver were 17.05 ± 10.40, 17.55 ± 10.86, and 15.04 ± 9.75 mSv, respectively. Table 3 and Figure 2 present the details of the relationship between SSDE and CTDI_vol_. Notably, SSDE values were higher than CTDI_vol_ for each group for both calculation methods. The deviation between SSDE and CTDI_vol_ narrowed as the subjects′ *D_W_* increased. In Figure 3, the effective cancer risk increases with age for lung and liver, but for breast, the effective cancer risk flattens at the age of 50 and is lower than lung when age was 70 years. Table 4 tabulates the cancer risk per million procedures with different organs and *D_W_*. Breast seemed to receive the highest organ dose in total, resulting in 94 future cancer risks per million procedures. All variables showed no significant difference between sexes except for *E*, as presented in Table 5. 

## 4. Discussion

The subjects′ size varied along the Z-axis of the scan due to changes in the thickness and composition of the subjects’ habitus. As expected, the variation in the subjects′ *D_W_* contributed to the SSDE in line with a previous study [14]. Hence, the CTDI_vol_ calculated from the console was observed to be undervalued compared to SSDE, especially in small-sized subjects. Overall, the reference phantom-based CTDI_vol_ values underestimated the radiation dose received by the subjects as compared to the SSDE approach. The small variations in the SSDE to CTDI_vol_ ratio generated by CT-Expo in different subject size groups were expected since the software′s calculations are based on a constant mathematical phantom [11]. However, the ratio generated by the AAPM report method was wider in small-sized groups compared to bigger-sized groups; the *f*-size increased as subjects’ body size decreased. As the previous study reported with the automated tube current modulation (ATCM) system, both CTDI_vol_ and SSDE values were higher in large-sized subjects, but without ATCM, both dose descriptors remained unchanged as body size increased [15]. This observation aligned with this study where the ATCM was deployed.

The use of low tube voltage was reported to be the most effective method to reduce the radiation dose exposure in CT examination, especially CTPA [16,17,18]. However, the low tube had to be applied cautiously without affecting image quality. Another study reported that reducing the tube voltage in CT examinations involving contrast media could maximize the photoelectric effect, as the applied voltage was closer to the K-edge of iodine (33.2 keV) [19]. Reducing the tube voltage could also enhance the performance of image quality and reduce the radiation dose. Most studies applied ATCM to modulate tube current and significantly reduced unnecessary exposure to patients [11,20,21,22,23,24]. However, different approaches, such as pitch factor selection and beam size collimation, have shown different radiation exposure outcomes with different institutions and CT-Scanner types.

Figure 3 illustrates the distribution of effective cancer risks. The breast and lungs receive the highest radiation dose exposure as these organs are within the primary beam. Both also had an equally high risk of developing cancer. The liver attained the lowest values in organ dose and cancer risk, mainly because, in a CTPA procedure, the liver is only be partially scanned as it is not entirely within the region of interest. This observation is in line with the BEIR VII report, which stated that dose exposure and cancer risk are dependent on the location of the organ relative to the primary beam, as well as the organ’s sensitivity to radiation. The higher tube current required to scan subjects with increasing body effective diameter was the main factor that caused the significant differences in organ doses and their cancer risk, as observed in Table 2 and Table 4. The ATCM system automatically modulates the tube current according to patient size and habitus [25,26]. Thus, subjects with large body sizes were at higher risk when undergoing CTPA. 

The liver had the lowest dose exposure even though the values increased with *D_W_*. However, its risk factor was extremely low—more than three times lower compared to the breast and lung. The breakdown of results between sexes is also provided. Table 5 shows that the *E*, organ doses, and cancer risk were all higher in women than men, but only the *E* was significant (*p* < 0.05). 

Unfortunately, the risk estimation in this study is not comparable with other studies, which presented various methodologies and different cases. Although not significant, the result of this study supports previous research that found a higher cancer risk in women. The overall lethality risk for women was approximately 35% higher in comparison with men, as illustrated in Table 5 [11]. There were some limitations to this study. Firstly, the subjects in this study were not adolescents; hence, further investigation is needed to evaluate the radiation dose and selected organ risk for pediatric age groups. Secondly, the SSDE, organ dose, and *E* values derived using CT-Expo software were not normalized to the *D_W_* of each patient. Thus, the values were not accurately estimated since the CT-Expo software only used a constant size mathematical phantom. However, this study overcame this limitation with another evaluation by AAPM report 220′s methods for SSDE calculation. Third, the value of cancer risk for selected organs was not accurately estimated due to the inaccurate value of organ dose derived using CT-EXPO. We did not use another approach to calculate the organ doses besides the CT-Expo for comparison purposes, which contributes to the limitation. Finally, we assessed only one center and one manufacturer′s scanner and may not represent other centers that use different scanner protocols and/or radiography practices. More research should be conducted on various scanners and hospitals to obtain more accurate radiation exposure and cancer risk levels in the patient population.

## 5. Conclusions

In this study, the effective risk associated with CTPA examinations was estimated regarding body diameter and sex. The results showed that the effective risk for male subjects is slightly lower than for female subjects. Most of the dose descriptors increased dramatically as *D_W_* increased. Notably, the SSDE values showed promise for accurately evaluating radiation dose, and radiation risks. The estimated cancer risk per million CTPA examinations of breast, lung, and liver organs were 0.009%, 0.007%, and 0.005%, respectively. This study shows that cancer risk differs significantly between body diameters of subjects.

## Figures and Tables

**Table 1:** Baseline characteristic and demography of the subjects.

Baseline Characteristic	Values		
	Male	Female	Total
Age (years/old) *	49.26 ± 14.57	48.60 ± 19.12	48.88 ± 17.28
Anteroposterior (AP) (cm) *	21.68 ± 3.68	21.88 ± 2.71	21.80 ± 3.14
Lateral (LAT) (cm) *	33.46 ± 4.17	32.85 ± 3.53	33.10 ± 3.80

* mean ± SD.

**Table 2:** Overview of the CTDI_vol_, DLP, E, and organ dose values from a different subject group′s *D_W_*_._

*D_W_* (cm)	Dose Descriptors *			Organ Equivalent Dose (mSv) *		
	CTDI_vol_ (mGy)	DLP (mGy cm)	E (mSv)	Breast	Lung	Liver
Group 1 (19–25)	6.44 ± 2.63	239.59 ± 97.36	5.19 ± 2.50	10.94 ± 4.62	10.62 ± 4.12	9.15 ± 4.23
Group 2 (25–28)	9.86 ± 6.46	351.85 ± 231.85	7.47 ± 4.11	15.48 ± 7.93	15.55 ± 8.39	13.48 ± 6.99
Group 3 (>28)	17.42 ± 6.90	631.46 ± 274.43	13.90 ± 5.66	23.81 ± 12.17	27.39 ± 11.87	23.19 ± 11.76
*p*-value	<0.05	<0.05	<0.05	<0.05	<0.05	<0.05
TOTAL	11.06 ± 7.17	400.38 ± 259.10	8.68 ± 5.47	17.05 ± 10.40	17.55 ± 10.86	15.04 ± 9.75

* mean ± SD.

**Table 3:** A comparison of SSDE values obtained from AAPM and CT-Expo with their ratios to CTDI_vol._

*D_W_* (cm)	Dose Descriptors			
	SSDE ^a^ (mGy)	SSDE ^b^ (mGy)	Ratio ^a^ SSDE/CTDI_vol_	Ratio ^b^ SSDE/CTDI_vol_
Group 1 (19–25)	9.93 ± 3.89	9.01 ± 3.78	1.54	1.30
Group 2 (25–28)	13.70 ± 9.04	13.41 ± 7.74	1.42	1.34
Group 3 (>28)	22.29 ± 7.35	23.98 ± 9.63	1.28	1.31
TOTAL	14.62 ± 8.41	15.37 ± 9.67	1.41	1.32

^a^ SSDE = the value obtained from AAPM 220 report; ^b^ SSDE = the value obtained from CT-Expo calculator.

**Table 4:** Mean cancer risk estimation according to group study per one million procedures.

Cancer Risk (Per Million Procedures)	*D_W_* (cm)				*p*-Value
	Group 1 (19–25)	Group 2 (25–28)	Group 3 (>28)	Total	
Breast	46.34	91.76	136.34	93.82	<0.05
Lung	24.54	66.93	107.45	66.39	<0.05
Liver	16.44	49.10	70.87	45.94	<0.05

**Table 5:** Comparison based on different genders according to the CTPA examination.

Variable		Sex		*p*-Value
		Male	Female	
Total E (mSv) *		7.47 ± 4.11	9.53 ± 5.68	<0.05
Organ Dose (mSv) *	Breast	n/a	17.05 ± 10.40	n/a
	Lung	15.55 ± 8.39	17.63 ± 10.41	NS
	Liver	13.48 ± 6.99	14.58 ± 9.28	NS
Cancer Risk (per million procedures)	Breast	n/a	93.81	n/a
	Lung	50.31	78.03	NS
	Liver	38.86	51.07	NS

* mean ± SD, NS = not significant.
